# PHASED RETIREMENT IN OLDER AMERICAN WORKERS

**DOI:** 10.1093/geroni/igz038.462

**Published:** 2019-11-08

**Authors:** Shinae Choi, Genevieve Smith

**Affiliations:** The University of Alabama, Tuscaloosa, Alabama, United States

## Abstract

Older workers are engaging in “phased retirement” in which they transition from full-time working status to part-time working and eventually retire at a later age. This study investigated whether phased retirement was financially and psychologically beneficial for middle-aged and older adults in the United States. The current study examined data on financial and psychological well-being and retirement transitions (i.e., immediate retirement and phased retirement) from the Health and Retirement Study (HRS) at two time points, four years apart. We analyzed 5,106 middle-aged and older adults from the 2010 and 2014 waves of the HRS data set using chi-square and one-way analysis of variance tests. Our results showed that 66.8% of respondents remained full-time working, while 12.7% of respondents chose phased retirement and 15.2% of respondents jumped straight into retirement. Our findings suggest that phased retirement is beneficial for older Americans financially and psychologically. Specifically, the level of total household financial wealth was significantly higher for those who chose phased retirement than immediate retirees from workforce. In terms of psychological perspectives, immediate retirees experienced more depressive symptoms than those who chose phased retirement. Our findings could help individuals and households to be better equipped when preparing for retirement. Our findings could also provide a basis for further research into phased retirement and its impact on well-being in middle-aged and older Americans. Furthermore, policymakers could be better informed about retirement trends and create policies based on our findings to better help older individuals and households be financially and psychologically prepared for retirement.

